# Optimization of NaOH Molarity, LUSI Mud/Alkaline Activator, and Na_2_SiO_3_/NaOH Ratio to Produce Lightweight Aggregate-Based Geopolymer

**DOI:** 10.3390/ijms160511629

**Published:** 2015-05-21

**Authors:** Rafiza Abdul Razak, Mohd Mustafa Al Bakri Abdullah, Kamarudin Hussin, Khairul Nizar Ismail, Djwantoro Hardjito, Zarina Yahya

**Affiliations:** 1 Centre of Excellence Geopolymer & Green Technology (CEGeoGTech), School of Material Engineering, Universiti Malaysia Perlis (UniMAP), P.O. Box 77, D/A Pejabat Pos Besar, 01000 Kangar, Perlis, Malaysia; E-Mail: zarinayahya@unimap.edu.my; 2 Faculty of Engineering Technology, Uniciti Alam Campus, Universiti Malaysia Perlis, Sungai Chuchuh, 02100 Padang Besar, Perlis, Malaysia; E-Mail: vc@unimap.edu.my; 3 Faculty of Technology, Universitas Ubudiyah Indonesia, Jl. Alue Naga, Kec. Syiah Kuala Desa, Tibang, 23536 Banda Aceh, Indonesia; 4 School of Environmental Engineering, Universiti Malaysia Perlis, 01000 Jejawi, Perlis, Malaysia; E-Mail: nizar@unimap.edu.my; 5 Civil Engineering Department, Petra Christian University, Jalan Siwalankerto 121-131, Surabaya 60236, Indonesia; E-Mail: djwantoro.h@peter.petra.ac.id

**Keywords:** artificial lightweight aggregate, geopolymer, aggregate impact value, SEM, X-ray Diffraction (XRD), Fourier Transform Infrared (FTIR)

## Abstract

This paper presents the mechanical function and characterization of an artificial lightweight geopolymer aggregate (ALGA) using LUSI (Sidoarjo mud) and alkaline activator as source materials. LUSI stands for LU-Lumpur and SI-Sidoarjo, meaning mud from Sidoarjo which erupted near the Banjarpanji-1 exploration well in Sidoarjo, East Java, Indonesia on 27 May 2006. The effect of NaOH molarity, LUSI mud/Alkaline activator (LM/AA) ratio, and Na_2_SiO_3_/NaOH ratio to the ALGA are investigated at a sintering temperature of 950 °C. The results show that the optimum NaOH molarity found in this study is 12 M due to the highest strength (lowest AIV value) of 15.79% with lower water absorption and specific gravity. The optimum LUSI mud/Alkaline activator (LM/AA) ratio of 1.7 and the Na_2_SiO_3_/NaOH ratio of 0.4 gives the highest strength with AIV value of 15.42% with specific gravity of 1.10 g/cm^3^ and water absorption of 4.7%. The major synthesized crystalline phases were identified as sodalite, quartz and albite. Scanning Electron Microscope (SEM) image showed more complete geopolymer matrix which contributes to highest strength of ALGA produced.

## 1. Introduction

The high volume of LUSI (LU-Lumpur, SI-Sidoarjo) mud that began erupting near the Banjarpanji-1 exploration well in Sidoarjo, East Java, Indonesia needed to be converted into a useful and valuable alternative resource. This eruption affected an area of almost three square miles to a depth of 65 feet and thirty thousand people were displaced, costing Indonesia 3.7 billion dollars in damages and damage control. The total cost of damage and damage control reached 488 million US dollars (USD) in 2011 and increased to 730.7 million USD in 2014 [1,2,3,4,5,6,7].

During the last decade, considerable research efforts have been directed towards the development of inorganic geopolymers, due to the wide range of potential applications for these materials. Geopolymerization is an innovative technology that can transform several aluminosilicate materials into useful products called geopolymers or inorganic polymers [8,9]. The geopolymerization process for LUSI mud consists of a chemical reaction of the Si-Al mineral system in alkaline condition due to high Si and Al and low Ca content. The research to utilize LUSI mud as pozzolanic material were reported by Januarti and Triwulan [10], Nuruddin *et al.* [11], and Hardjito *et al.* [7]. There is no other published work yet studying the artificial lightweight aggregate using LUSI mud.

Most countries worldwide have taken into account the impact of earthquake forces in designing the lightweight structure [12,13]. Lightweight concrete can reduce the mass of the structure and overall cost of the building. By combining these two technologies, the artificial lightweight aggregate using the geopolymer system is one of the solutions used to solve the abundance of the LUSI mud. Thus, the the current study is focused on the production of the optimum artificial lightweight geopolymer aggregate (ALGA) using LUSI mud and alkaline activator. The main objective of this paper is to study the effect of NaOH molarity, LUSI mud/Alkaline activator (LM/AA) ratio, and Na_2_SiO_3_/NaOH ratio to the mechanical function and characterization of ALGA produced.

## 2. Results and Discussion

### 2.1. Specific Gravity

**Figure 1:**
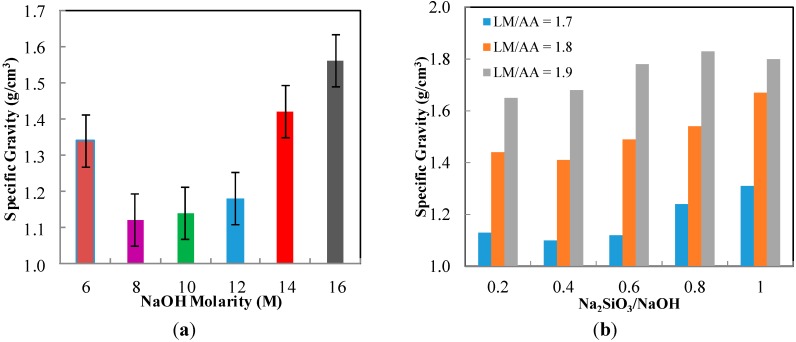
The specific gravity of ALGA at (**a**) various NaOH molarity; (**b**) various LUSI mud/Alkaline activator (LM/AA) ratio and Na_2_SiO_3_/NaOH ratio.

The specific gravity of ALGA produced at various NaOH molarity is shown in Figure 1a. The lowest specific gravity is observed at 8 M NaOH molarity with 1.12 g/cm^3^. After 8 M, the specific gravity of ALGA is higher with increasing NaOH molarity. However, high specific gravity was also detected at 6 M NaOH molarity due to insufficient concentration of NaOH to be reacted with LUSI mud particles. The specific gravity of ALGA at 8, 10, and 12 M are merely different with 1.12 g/cm^3^, 1.14 and 1.18 g/cm^3^, respectively. The high specific gravity is detected at 14 and 16 M due to the exceeded concentration of NaOH.

The specific gravity of ALGA produced at various LUSI mud/Alkaline activator (LM/AA) ratio and Na_2_SiO_3_/NaOH ratio are shown in Figure 1b. It is clearly shown that the higher LM/AA ratio gives higher specific gravity of ALGA produced. At Na_2_SiO_3_/NaOH ratio of 0.4 showed decreases of specific gravity, then kept increasing after 0.4 for all types of LM/AA ratio. The lowest specific gravity is observed at LM/AA ratio of 1.7 and Na_2_SiO_3_/NaOH ratio of 0.4 with 1.10 g/cm^3^. This value is merely different with ALGA produced at Na_2_SiO_3_/NaOH ratio of 0.2 and 0.6 with 1.13 and 1.12 g/cm^3^, respectively. For LM/AA ratio of 1.8 and 1.9, insignificant results were observed due to high specific gravity produced.

### 2.2. Water Absorption

**Figure 2:**
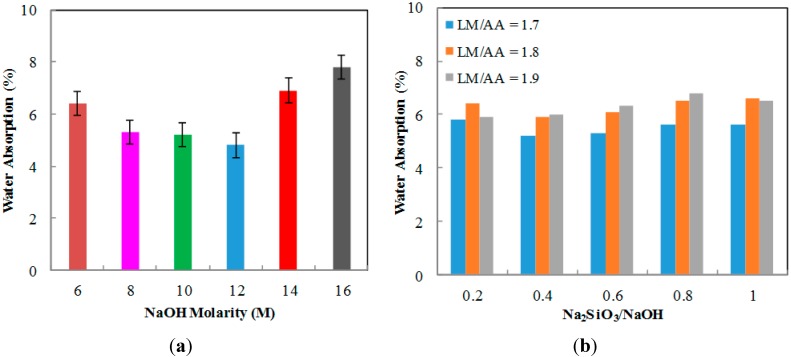
The water absorption of ALGA at (**a**) various NaOH molarity; (**b**) various LUSI mud/Alkaline activator (LM/AA) ratio and Na_2_SiO_3_/NaOH ratio.

Figure 2a shows the graph of water absorption of ALGA produced at various NaOH molarity. The highest water absorption is found at 16 M·NaOH molarity with 7.8% and the lowest water absorption is found at 12 M NaOH molarity with 4.8%. This is due to sufficient concentration of NaOH to be reacted with LUSI mud, hence, producing ALGA with optimum reaction while creating more uniform pores or voids inside the ALGA. However, the water absorption at 8, 10, and 12 M showed slight differences compared to 6, 14, and 16 M. The high molarity of NaOH will create the pores or voids at a larger size, which then creates more space to absorb the water, hence, increasing the water absorption results. Figure 2b shows the graph of twenty-four hour water absorption of ALGA produced at various LM/AA ratio and Na_2_SiO_3_/NaOH ratio. There is no specific pattern for water absorption produced. The highest water absorption is found at LM/AA ratio of 1.9 and Na_2_SiO_3_/NaOH ratio of 0.8 with 6.8% and the lowest water absorption is found at LM/AA ratio of 1.7 and Na_2_SiO_3_/NaOH ratio of 0.4 with 4.7%.

### 2.3. Aggregate Impact Value (AIV)

**Figure 3:**
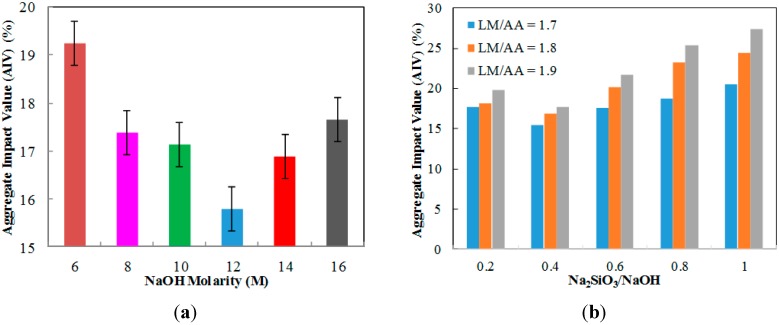
The aggregate impact value (AIV) of ALGA at (**a**) various NaOH molarity; (**b**) various LUSI mud/Alkaline activator (LM/AA) ratio and Na_2_SiO_3_/NaOH ratio.

Figure 3a shows the impact value of ALGA at various NaOH molarity. The lowest AIV was found at 12 M NaOH molarity with 15.79%. The highest AIV is observed at 6 M NaOH molarity with 19.25% indicating lower strength compared to ALGA produced at 12 M NaOH molarity. This is likely due to the lower dissolution ability of LUSI mud at low NaOH molarity, thus causing insufficient Na^+^ ions to allow for complete polymerization of the network [14]. The best AIV value was detected at ALGA produced at 12 M NaOH molarity due to lowest AIV value produced. This is due to sufficient Al^3+^ and Si^4+^ ions released from the alumina-silicates to participate in geopolymerization process with the optimum alkalinity medium. Sufficient alkali must be present for the complete dissolution process, then giving an increased geopolymerisation rate and higher strength [15]. The AIV of ALGA produced at high molarity of 14 and 16 M is increasing after 12 M NaOH molarity. This showed that the high molarity of NaOH does not necessarily give high strength of ALGA produced. Even though higher NaOH molarity has a higher rate of dissolution, it is however not desired by the polycondensation process [16] as excess Na+ ions left in the system then weaken the structure [14].

Figure 3b shows the AIV of ALGA at various LM/AA ratio and at the Na_2_SiO_3_/NaOH ratio. The lowest AIV was found at the LM/AA ratio of 1.7 and Na_2_SiO_3_/NaOH ratio of 0.4 with 15.42%. The highest AIV is observed at LM/AA ratio of 1.9 and Na_2_SiO_3_/NaOH ratio of 1.0 with 27.40%, indicating lower strength. High LM/AA ratio indicates low workability and this caused difficulty during the palletizing process where failure in providing good compaction may reduce the strength of the ALGA produced. This statement also proved by Kong *et al.* [17] which concludes the same reason for cement paste. All graphs show the same pattern with high AIV value at a Na_2_SiO_3_/NaOH ratio of 0.2 then decreased at a 0.4 ratio. After that, the AIV value keeps increasing at a Na_2_SiO_3_/NaOH ratio of 0.6, 0.8 and 1.0 for all LM/AA ratio samples. For low Na_2_SiO_3_/NaOH ratio of 0.2, the excess of Na+ ions was believed to exist in structure, which will form the sodium carbonate, then affect the geopolymerization process and resulted in lower strength. For high Na_2_SiO_3_/NaOH ratio, excess of Na_2_SiO_3_ may hinder the evaporation of water and structure formation [18]. The best LM/AA ratio of 1.7 and the Na_2_SiO_3_/NaOH ratio of 0.4 is believed to provide the optimum rate of geopolymerization process due to the highest strength (lowest AIV value) with lower water absorption and specific gravity. The correct proportion and spatial distribution of an aluminosilicate network will maximize the mechanical strength of the geopolymer matrix [19].

### 2.4. X-ray Diffraction (XRD)

**Figure 4:**
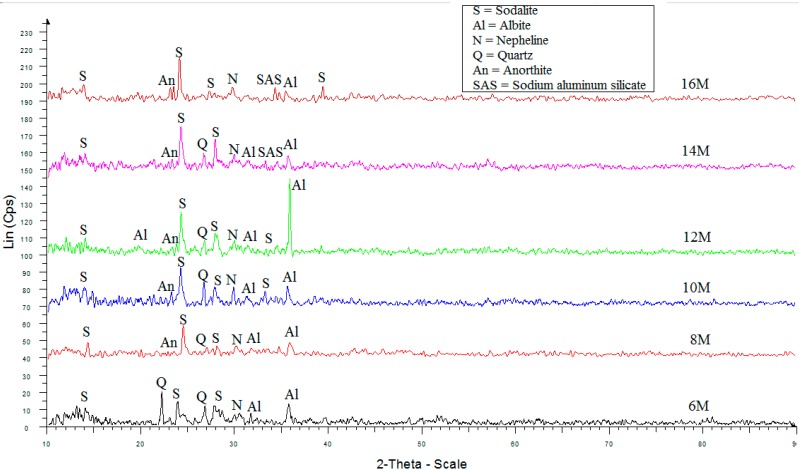
XRD pattern of ALGA produced at various NaOH molarity.

XRD analysis of ALGA from LUSI mud at various NaOH molarity are shown in Figure 4. The major synthesized crystalline phases were identified as sodalite (Na_4_Al_3_Si_3_O_12_Cl), albite (NaAlSi_3_O_8_) and quartz. The broad humps were detected at 2θ = 21°–36°, suggesting that amorphous phases are present showing the geopolymerization was occurred. High intensity has been detected at 2θ = 22.2° at 6 M indicating quartz, showing it did not take part in geopolymerization process. Then the high intensities observed at 2θ = 24.6° at others NaOH molarity which are all associated to the new phase of sodalite (S) formed. However the highest intensity peak of 12 M was found at 2θ = 35.8° which indicating the high formation of albite (Al) peak appeared. According to Eun Oh *et al.* [20,21], the strength forming phases which forms the crystalline phases was assigned to albite. This high intensity showed the sufficient reaction of the LUSI mud introduced in the alkaline activator. The new phases of albite (Al) was also detected at 2θ = 21.0°, thus showing this new phase contribute to the high strength of ALGA produced at 12 M. The sodium aluminium silicate (SAS) appeared at ALGA produced at 14 and 16 M which was associated with the efflorescence presented in SEM results caused by excess sodium oxide remaining unreacted in the material and contribute to low strength of ALGA produced.

The nepheline (Na_3_KAl_4_Si_4_O_16_) peaks has were detected at 2θ = 31° at 6 and 8 M, then shifted to the left at 2θ = 30.0° at 10, 12, 14 and 16 M. The anorthite (CaAl_2_Si_2_O_8_) phase also detected at 2θ = 23.0° at all molarities, except at 6 M. The presence of anorthite phase indicates the reaction between calcium from the LUSI mud with alumina-silicate. Exception of anorthite at 6 M was due to low concentration of NaOH to be reacted with calcium in LUSI mud.

**Figure 5:**
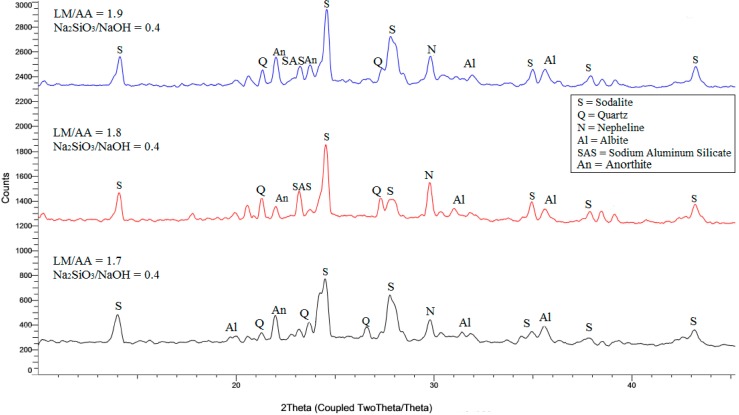
XRD pattern of ALGA produced at various LM/AA ratio and Na_2_SiO_3_/NaOH ratio.

Meanwhile, the XRD analysis of ALGA produced from LUSI mud at the best three mix design LM/AA ratio and Na_2_SiO_3_/NaOH ratio is shown in Figure 5. Sodalite still remained as the highest intensity for each sample, but the intensities of sample at LM/AA of 1.7 and Na_2_SiO_3_/NaOH of 0.4 was the lowest due to the dilution effect into more stable alkaline aluminosilicates. For LM/AA ratio of 1.7 and the Na_2_SiO_3_/NaOH ratio of 0.4, the major synthesized crystalline phases were identified as sodalite (S) of Na_4_Al_3_Si_3_O_12_Cl, quartz (Q), albite (Al), anorthite (An) and nepheline (N) that strengthen the ALGA produced [2,22]. The broad humps were detected at 2θ = 21°–36°, suggesting that amorphous phases are present showing the geopolymerization occurred. The highest intensity has been detected at 2θ = 24.6° for all samples which associated to sodalite (S). For LM/AA ratio of 1.8 and the Na_2_SiO_3_/NaOH ratio of 0.4, the major phases were sodalite (S), sodium aluminium silicate (SAS), anorthite (An) and albite (Al).

For LM/AA ratio of 1.9 and Na_2_SiO_3_/NaOH ratio of 0.4, the major phases were sodalite (S), sodium aluminium silicate (SAS), anorthite (An), and albite (Al). The crystalline phase of sodalite also has been found by Criado *et al.* [23] and Alvarez-Ayuso *et al.* [24] for fly ash-based geopolymer. Sodalite framework structure is a rigid structure and more flexible at the larger unit cell [21], thus contributing to the strength of the structure. The existence of sodium aluminum silicate (SAS) at LM/AA ratio of 1.8 and 1.9 showed the existence of efflourescence which then contributes to low strength as proved by AIV results.

### 2.5. Scanning Electron Microscope (SEM)

**Figure 6:**
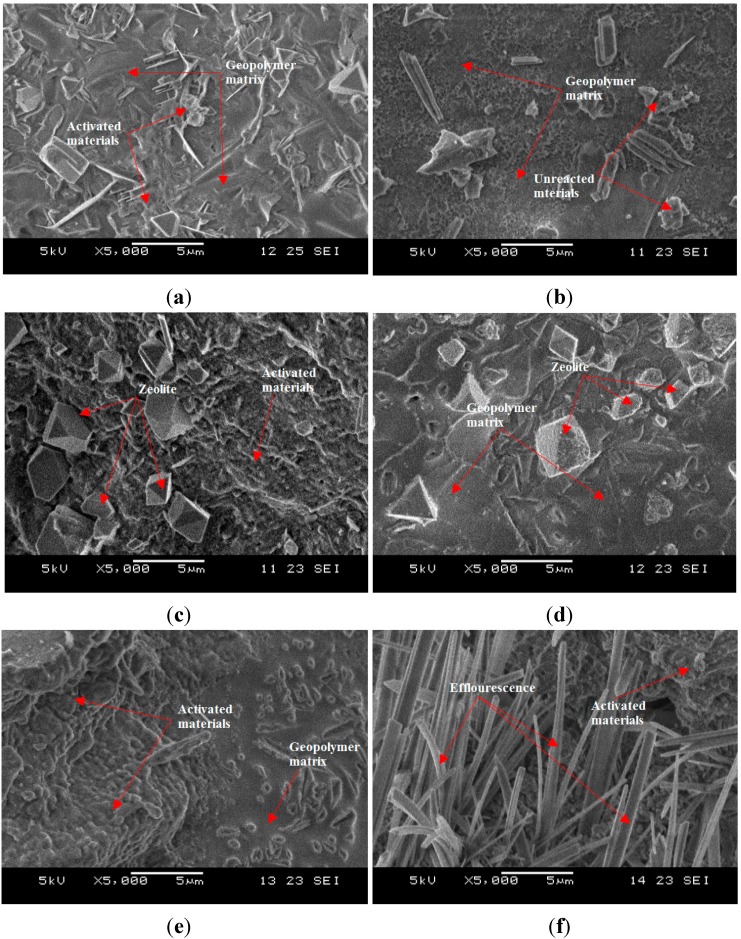
SEM micrograph of ALGA produced at various NaOH molarity, (**a**) 6 M; (**b**) 8 M; (**c**) 10 M; (**d**) 12 M; (**e**) 14 M; and (**f**) 16 M.

Figure 6 shows the SEM images of ALGA produced at various NaOH molarity at the sintering temperature of 950 °C. All samples showed the existence of pores that contribute to lightweight structure. Some unreacted particles precipitate after water evaporates during curing at 6 and 8 M due to less molarity of NaOH to be reacted with LUSI mud particles. Microstructure of ALGA produced at 6, 8, and 10 M consist of discrete prism-shaped particles, a few at 12 M, while the other shows a continuous gel-like matrix at 10 and 14 M. The continuous gel-like area with some voids at 12 M is made of pure geopolymer binder which strengthens the structure itself and contributes to high strength. The presence of zeolite phase (sodalite) at 10 and 12 M showed by cubic morphology as shown in Figure 6a,b.

The alkaline activation of LUSI mud caused the dissolution of silicate and aluminate species into solution. The dissolved alumina (Al) will firstly react with the silicate (from sodium silicate) to form the silicate oligomers, then grows and begins to crystallize forming zeolites [25].

The presence of efflorescence was observed on the samples ALGA of 16 M (Figure 6f). According to Temuujin *et al.* [26], the efflorescence is the indication of insufficient geopolymerisation reaction or excess alkali. In this case, the ALGA produced at the high concentration of 16 M showed the existence of efflorescence due to excess alkali content. The excess alkaline is not immobilized within the geopolymer structure and then, it will be expelled from the geopolymer as sodium phosphate as suggested by Davidovits [27].

ALGA with NaOH molarity 14 and 16 M showed the more dense structure due to excessive concentration of NaOH in geopolymer matrix. These contribute to the high density of ALGA produced, but low strength due to the presence of efflorescence.

**Figure 7:**
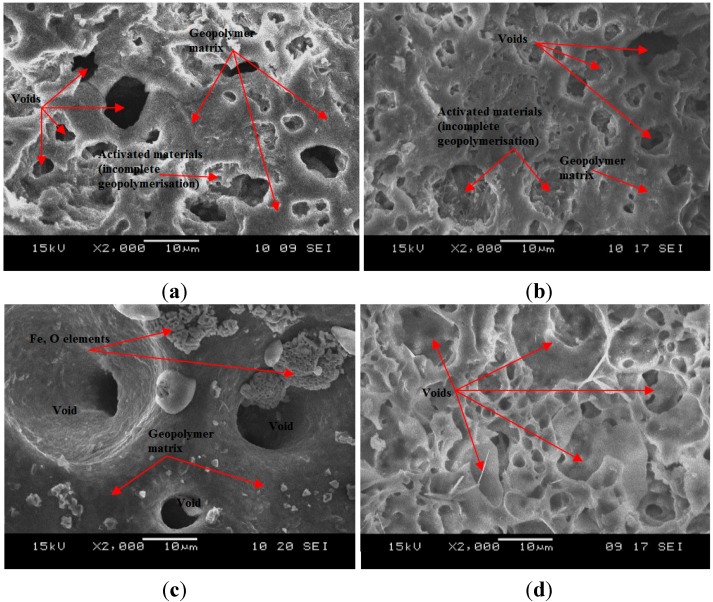
SEM micrograph of ALGA produced at (**a**) LM/AA = 1.7 and Na_2_SiO_3_/NaOH = 0.4; (**b**) LM/AA = 1.8 and Na_2_SiO_3_/NaOH = 0.4; (**c**) LM/AA = 1.9 and Na_2_SiO_3_/NaOH = 0.4; (**d**) control samples.

The SEM micrographs of ALGA produced at the best three mix design (high strength) of LM/AA ratio with Na_2_SiO_3_/NaOH ratio and control samples (only LUSI mud-without binder of alkaline activator) are shown in Figure 7. At LM/AA ratio of 1.7 and Na_2_SiO_3_/NaOH ratio of 0.4 (Figure 7a) showed more complete geopolymer matrix which contributes to highest strength of ALGA produced. The voids formed were in the range of 2.5–6.9 µm in diameter, which give satisfied specific gravity produced for ALGA (1.10 g/cm^3^).

For ALGA produced at LM/AA ratio of 1.8 and the Na_2_SiO_3_/NaOH ratio of 0.4 (Figure 7b) showed more dense and continuous gel-like of geopolymer matrix with voids size of 3.2–12.4 µm. Some part showed the needle or lathe-shaped particles are formed in the voids surface indicating activated materials. In addition to the major elements (Si, Al, Na, O) making up the geopolymer, high composition of Fe is also present as impurities. Those needle-shaped particles are believed come from Fe elements as proved by EDX spectrum. According to Duxson *et al.* [28], this Fe element has some influence on the geopolymerization process.

For ALGA produced at LM/AA ratio of 1.9 and Na_2_SiO_3_/NaOH ratio of 0.4 (Figure 7c) showed large voids with a size range of 15.2–20.0 µm. The presence of large voids is due to low workability during the palletization process, thus failure to compact well, which then provide voids. It can be seen that the Fe elements exist at the surface structure which showed that this element was not taking part in the geopolymerisation process. This is due to high contents of LUSI mud from LM/AA ratio of 1.9. High LM/AA ratio does not produce significant effects on the strength of ALGA produced.

Figure 7d showed the SEM micrograph of control samples (LUSI mud only without alkaline activator). The matrix formed is clearly thinner compared to ALGA produced as shown in Figure 7a. This thinner matrix may contribute to the low strength of the aggregate produced. The pores presented in the control samples are higher than ALGA produced with sizes range of 1.5–12.6 µm. It can be concluded that the presence of an alkaline activator as a binder to LUSI mud, making the structure to be more stable with thicker matrix formed, which then contribute to the high strength of ALGA produced.

### 2.6. Fourier Transform Infrared

**Figure 8:**
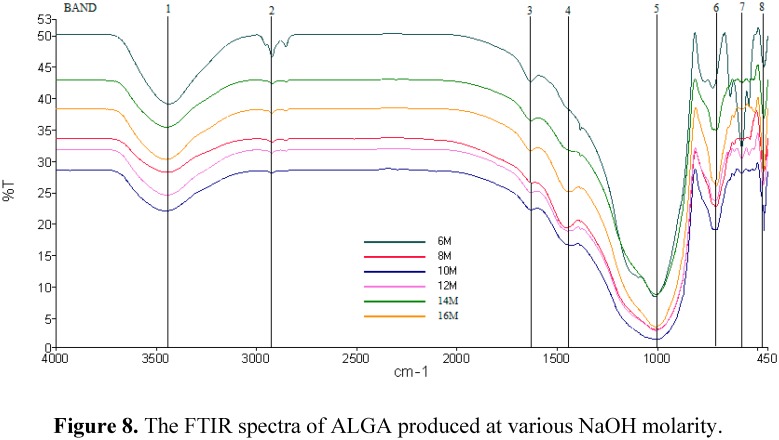
The FTIR spectra of ALGA produced at various NaOH molarity.

Figure 8 shows the Fourier Transform Infrared *(*FTIR) spectra of ALGA produced at various NaOH molarity. Transformation took place during synthesis of geopolymer was indicated by the different absorption frequencies of ALGA produced. The O–C–O stretching vibration (band 4) exists in the ALGA produced at 12, 14, and 16 M at 1431–1450 cm^−1^, but is absent at low molarity of 6, 8, and 10 M. The asymmetric stretching vibrations of Si-O-Si and Al-O-Si at 1012 cm^−1^ (band 5) for 6, 8, and 10 M are the same, but then shifted to the lower frequency of 1007 cm^−1^ at 12 M, then turn to 1011 cm^−1^ at 14 and 16 M. This indicates the formation of a new product of sodium aluminum silicate (SAS) phase as proved by XRD results, which is associated with the higher dissolution of LUSI mud in the strong molarity of alkaline activator solution (12, 14, and 16 M).

The Al–O–Si stretching vibrations (band 6) appeared in all samples at 6–16 M. Moreover, the structural reorganization of LUSI mud is evidenced by the appearance of band 7 at lower frequencies (579 cm^−1^) assigned to symmetric stretching vibrations of Si–O–Si and Al–O–Si [29]. Band 8 located at 467–471 cm^−1^ appeared at all ALGA produced at 6–16M, which assigned to bending vibrations of Si–O–Si and O–Si–O bonds [29,30,31].

**Figure 9:**
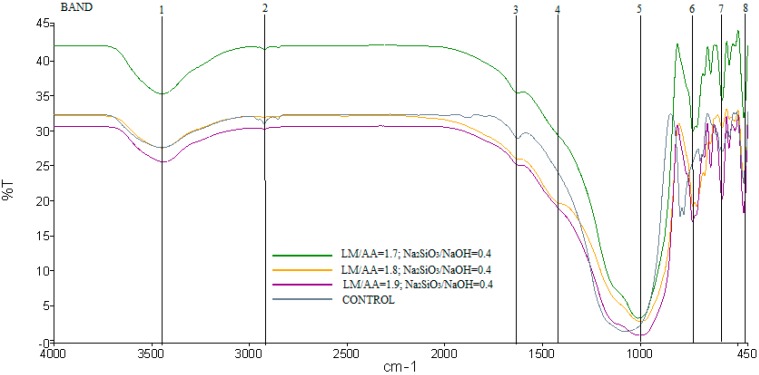
FTIR spectra of ALGA produced at LM/AA ratio of 1.7, 1.8, and 1.9 with Na_2_SiO_3_/NaOH ratio of 0.4 and control sample.

Figure 9 shows the FTIR spectra of ALGA produced at the best three mix design of LM/AA ratio with Na_2_SiO_3_/NaOH ratio and control samples (only LUSI mud-without alkaline activator). The bands 1, 2, and 3 at 1627–1630 and 2921–3451 cm^−1^ arise from the presence of structural water remaining in the geopolymer matrix of ALGA and control sample. The most characteristic difference observed between the FTIR spectrum of ALGA and control sample is the band 5 of asymmetric stretching vibrations of Si–O–Si and Al–O–Si. The band of 1075 cm^−1^ in the FTIR spectrum of aggregate produced without alkaline activator (control samples), then shifts to lower frequencies (995–1012 cm^−1^) in the FTIR spectra of ALGA produced at LM/AA ratio of 1.7, 1.8 and 1.9 with Na_2_SiO_3_/NaOH ratio of 0.4. Verdolotti *et al.* [32] stated that the broadness of the absorbance band 5 at 820–1250 cm^−1^ showed the variability of the bond angles and bond lengths of the tetrahedral structures around the silicon atoms.

The absorbance peak at 731 cm^−1^ (band 6) that appeared at ALGA produced the best mix designs which were assigned to stretching vibration of Al–O–Si bonds [29,31], but absent in the control samples. The bending vibration of Si–O–Si and O–Si–O can be seen in all samples at band 8 as shown in Figure 9.

## 3. Experimental Section

### 3.1. Source Materials and Treatment

#### 3.1.1. LUSI Mud

The present study was conducted on the LUSI mud collected from the eruption sites near Sidoarjo, East Java, Indonesia. The received batches of LUSI mud were already partially dried. The LUSI mud needs to be treated (dried at 105 °C, crushed and sieved passing a 300 µm sieve) before transforming into the ash form to simplify the palletizing process of aggregate and remove any other coarser particles. The chemical composition of LUSI mud is done by X-ray Flourescence (XRF) and the details are shown in Table 1. The total of SiO_2_ + Al_2_O_3_ + Fe_2_O_3_ >70% indicated that this LUSI mud can be used as pozzolan materials (ASTM C-618) [33] and is suitable to be used as a raw material for geopolymer.

**Table 1:** Chemical composition of LUSI mud.

Component	Al_2_O_3_	SiO_2_	K_2_O	TiO_2_	Fe_2_O_3_	CaO	MnO	SO_3_	V_2_O_5_	LOI
LUSI mud (%)	14.60	40.00	4.28	1.75	23.25	5.46	0.34	0.88	0.06	9.38

**Figure 10:**
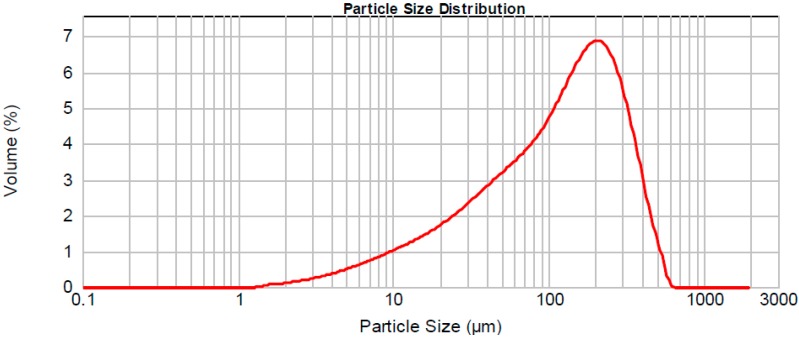
Graph of particle size distribution of LUSI mud.

The mean particle size of the LUSI mud is dominated by particles in the size d (0.5) of 121 µm with specific surface area of 0.151 m^2^/g. Finer particles provide high surface area to react in geopolymerization process and affect the strength of geopolymer. LUSI mud needs to be in fine particles to simplify the mixing process of geopolymer mixture in the production of ALGA. Figure 10 shows the particle size distribution of LUSI mud.

**Figure 11:**
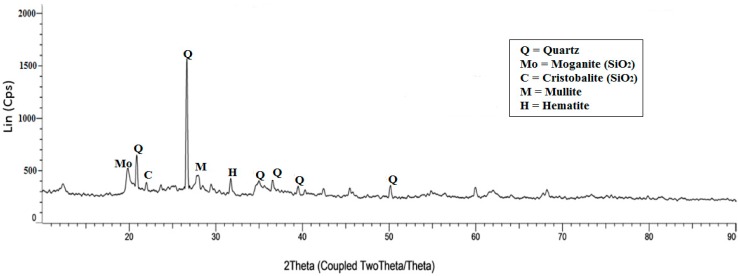
X-ray Diffractogram of original LUSI mud.

An X-ray diffraction (XRD) analysis of LUSI mud is shown in Figure 11. LUSI mud is dominated by amorphous phase with crystalline peak of quartz and silicon dioxide (SiO_2_). This material exhibits its highest peak at 2θ = 26.6° due to higher intensity of quartz (Q) (SiO_2_). This was also demonstrated by the XRF results which shows that the higher contents of SiO_2_ represented with high peak of intensity in XRD results. LUSI mud showed two other intense diffraction peaks at 2θ = 21.0°, 35.0°, 36.6°, 39.2° and 50.2°, which are associated with quartz (Q). The 2θ values of 27.9° revealed the mullite and 31.8° revealed the hematite (Fe_2_O_3_) element. The moganite (M) and crystobalite (C) were also detected at 2θ = 19.7° and 21.8°, respectively.

**Figure 12:**
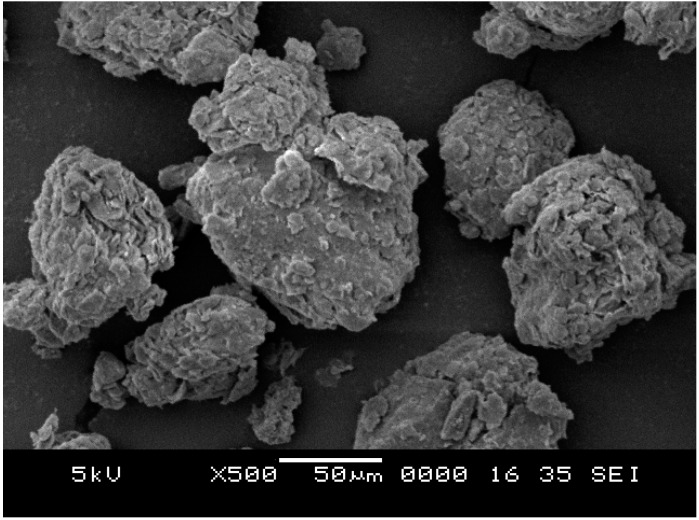
SEM images of original LUSI mud.

LUSI mud has a plate-like structure which was similar to kaolin as shown in Figure 12. The structure of original LUSI mud shows more layers stick together to form the bigger structure due to the existence of water. The shape at high magnification of 500× of LUSI mud showed agglomerates of irregular shape, like a tissue texture that has been torn depending on the crushed and blended process during sample preparation.

**Figure 13:**
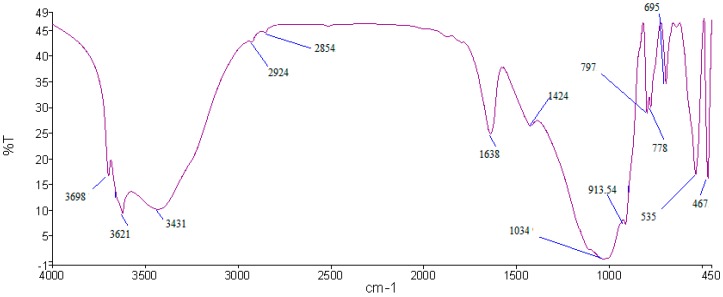
FTIR spectrum of LUSI mud.

Figure 13 shows that the FTIR adsorption bands of the LUSI mud. LUSI mud showed characteristic peaks at 3698, 3621 and 3431 cm^−1^, corresponding to the OH- stretching vibration. The OH– and H–O–H stretching vibration are assigned at 2924 and 2854 cm^−1^. The H–O–H bending at 1638 cm^−1^ assigned to the weakly-bound water molecules, which are adsorbed on the surface or trapped in the large cavities between the agglomerated LUSI mud. The stretching vibration of O–C–O is assigned at 1424 cm^−1^. Band at 1034 cm^−1^ was assigned to Si-O bonds in the SiO_4_ molecules. The bands at 797 and 778 cm^−1^ are assigned to Si–O–Si symmetric stretching. The band at 695 cm^−1^ is assigned to Al–O–Si bending, meanwhile the band at 467 cm^−1^ is assigned to Si–O–Si and O–Si–O bending.

#### 3.1.2. Alkaline Activator

Sodium hydroxide (NaOH) used in this study was from the pallet type of Formosoda-P from Formosa Plastic Corporation, Taiwan. Sodium silicate used was obtained from South Pacific Chemical Industries Sdn. Bhd. (SPCI), Malaysia. Sodium silicate has a chemical composition of 30.1% SiO_2_, 9.4% Na_2_O and 60.5% H_2_O (modulus SiO_2_/Na_2_O = 3.2), specific gravity at 20 °C = 1.4 g/cm^3^ and viscosity at 20 °C = 0.4 Pa∙s.

### 3.2. ALGA Preparation

In order to find the effect of NaOH molarity to the ALGA, the ratio of LUSI mud/Alkaline activator (LM/AA) also should be fixed to 1.7 for all mixtures. The mass of LUSI mud and alkaline activator solution were kept constant for all 6, 8, 10, 12, 14 and 16 M [34,35] with various molarity. The mass of NaOH pallet used for each type of molarity is shown in Table 2. Then, all samples were sintered at sintering temperature of 950 °C [36].

**Table 2:** Detail preparation of NaOH solution.

NaOH Molarity (M)	The Mass of the NaOH Pallet to Be Dissolved in One Litre (1 L) Distilled Water (g)
6	240
8	320
10	400
12	480
14	560
16	640

After finding the best NaOH molarity, the optimum mix design on producing ALGA should be studied. The details of the mix design of various ratios of LM/AA and Na_2_SiO_3_/NaOH are shown in Table 3.

**Table 3:** Details of mix design for various ratios of LUSI mud/alkaline activator (LM/AA) and Na_2_SiO_3_/NaOH.

Ratio of LM/AA	Ratio of Na_2_SiO_3_/NaOH	Mass of LUSI Mud (g)	Mass of Na_2_SiO_3_ (g)	Mass of NaOH (g)
1.7	0.2	1511	333	556
	0.4		485	404
	0.6		571	317
	0.8		627	261
	1.0		667	222
1.8	0.2	1543	321	536
	0.4		468	390
	0.6		551	306
	0.8		605	252
	1.0		643	214
1.9	0.2	1572	310	517
	0.4		451	376
	0.6		532	296
	0.8		584	243
	1.0		621	207

In this experiment, the ratio of LM/AA used were 1.7, 1.8 and 1.9. These values were chosen due to the workability during the palletizing process to form the aggregate. The workability is too high if LM/AA below 1.7 is used, meanwhile the workability is too low if LM/AA above than 1.9 is used. The ratio of Na_2_SiO_3_/NaOH used are 0.2, 0.4, 0.6, 0.8, and 1.0. The best mix design ratios then compared with the control sample (artificial lightweight using LUSI mud only without alkaline activator).

### 3.3. Mixing, Palletizing and Sintering Process

Alkaline activator solution (mixture of Na_2_SiO_3_ and NaOH solution) needs to be prepared for 24 h prior to use. After all materials have been prepared and weighted, the geopolymer paste is prepared by mixing LUSI mud that has been treated to ash form with the alkaline activator for 10 min by using a hand mixer, forming a homogeneous paste.

The fresh paste need to be palleted to a round shape with range size of 10–14 mm in diameter to be standard as normal aggregate. No extra water that should be added during the palletizing process. The pallets are then dried at the temperature 60 °C [37] for 30 min to obtain the shape of dried aggregate before sintering process. This drying process is needed to prevent cracking and exploding of the pellets if wet pellets are used [38]. The palletizing process was done by hand manually to control the target size of pallets produced.

Furnace model Carbolite CWF 1200 has been used for sintering purposes. This furnace has a working temperature up to 1100 °C. Sintering is a very significant process in order to achieve ALGA of high strength with low density. The sintering process at 950 °C is the hardening process with “light” aggregate produced. In this study, the increment rate of temperature from room temperature up to 950 °C was 16 °C per minutes. Then, the soaking time of 950 °C was maintained for 1 h. After a soaking time of 1 h, the furnace will be cooled down to room temperature without control. Normally it takes about 8 to 10 h to be cooled to room temperature.

### 3.4. Testing Methods

#### 3.4.1. Specific Gravity

Specific gravity is a measure of density relative to the density of a reference substance. The specific gravity of samples is conducted by using Electronic Densimeter MD-3005 from Alfa Mirage (Bangkok, Thailand).

#### 3.4.2. Water Absorption

The water absorption test has been conducted according to ASTM C140 [39] to obtain the water absorbed by ALGA that is immersed in water.

#### 3.4.3. Aggregate Impact Value (AIV)

Aggregate impact value (AIV) test was done to determine the impact value of artificial geopolymer aggregate from LUSI mud as per MS30:PART 10:1995. The value of AIV represents the strength of aggregate produced.

#### 3.4.4. X-ray Diffraction (XRD)

X-ray diffraction (XRD) patterns were obtained by using X-ray diffractometer XRD-6000 (Shimadzu, Tokyo, Japan) using Cu-Kα radiation generated at 30 Ma and 40 kV. The XRD test should be carried out to get the phase analysis of ALGA produced.

#### 3.4.5. Scanning Electron Microscope (SEM)

Morphology of LUSI mud for ALGA produced were revealed by using JSM-6460LA model Scanning Electron Microscope (JEOL) (Tokyo, Japan). In this testing, the samples were cut into fine and smooth small pieces portion. Then, all specimens were coated with palladium by using Auto Fine Coater JEOL JFC 1600 model before the testing.

#### 3.4.6. Fourier Transform Infrared (FTIR)

Fourier transform infrared (FTIR) spectroscopy analysis of ALGA of different parameters are performed by using Perkin Elmer FTIR Spectrum RX1 Spectrometer. The samples are prepared in powder form and KBr pellet technique. The samples were scanned from 500 to 4000 cm^−1^ with resolution of 4 cm^−1^.

## 4. Conclusions

The optimum NaOH molarity found in this study is 12 M due to the highest strength (lowest AIV value) of 15.79% with lower water absorption and specific gravity to be performed on ALGA lightweight concrete. The continuous gel-like area with some voids at 12 M is made of pure geopolymer binder which strengthens the structure itself and contributes to high strength. The new phases of albite (Al) (NaAlSi_3_O_8_) and sodium aluminium silicate (SAS) appeared at ALGA produced at 12 M which might affect the highest strength produced.

For selection of optimum mix design, the LUSI mud/Alkaline activator (LM/AA) ratio of 1.7 and Na_2_SiO_3_/NaOH ratio of 0.4 give the highest strength with AIV value of 15.42% with specific gravity of 1.10 g/cm^3^ and water absorption of 4.7%. The major synthesized crystalline phases were identified as sodalite (S), silicon dioxide (SiO_2_) and quartz (Q). SEM image showed more complete geopolymer matrix which contributes to highest strength of ALGA produced. The band of 1075 cm^−1^ assigned to asymmetric stretching vibrations of Si–O–Si and Al–O–Si in the FTIR spectrum of control sample shifts to lower frequencies (~995–1012 cm^−1^) in the FTIR spectra of ALGA produced at the best mix design. The broadness of the absorbance band at 820–1250 cm^−1^ showed the variability of the bond angles and bond lengths of the tetrahedral structures around the silicon atoms.

The performance of ALGA can be further analyzed in lightweight concrete application for the future works.
